# Immunomodulatory Effects of *Cucurbita pepo* L. Extract in Chronic Stress-Induced Dysregulation of Lymphoid Organs in Rats

**DOI:** 10.3390/ph18071046

**Published:** 2025-07-17

**Authors:** Safa H. Qahl, Hailah M. Almohaimeed, Sami A. Algaidi, Ashwaq H. Batawi, Zuhair M. Mohammedsaleh, Tarek Hamdy Abd-Elhamid, Nawal H. Almohammadi, Nasra N. Ayuob, Amany Refaat Mahmoud

**Affiliations:** 1 Department of Biological Sciences, College of Science, University of Jeddah, P.O. Box 80327, Jeddah 21589, Saudi Arabia; shqahal@uj.edu.sa; 2 Department of Basic Science, College of Medicine, Princess Nourah bint Abdulrahman University, P.O. Box 84428, Riyadh 11671, Saudi Arabia; hmalmohaimeed@pnu.edu.sa; 3 Department of Basic Medical Science, Faculty of Medicine, Taibah University, P.O. Box 344, Madinah 42353, Saudi Arabia; sqaidi@taibahu.edu.sa (S.A.A.); nhmohammadi@taibahu.edu.sa (N.H.A.); 4 Department of Biological Science, Faculty of Science, King Abdulaziz University, Jeddah 21589, Saudi Arabia; abatawi@kau.edu.sa; 5 Department of Medical Laboratory Technology, Faculty of Applied Medical Sciences, University of Tabuk, Tabuk 71491, Saudi Arabia; zsaleh@ut.edu.sa; 6 Department of Histology and Cell Biology, Faculty of Medicine, Assiut University, Assiut 71515, Egypt; tabdelhamid@aun.edu.eg; 7 Department of Basic Medical Sciences, Faculty of Medicine, Aqaba Medical Sciences University, Aqaba 77110, Jordan; 8 Department of Medical Histology, Faculty of Medicine, Damietta University, Damietta 34511, Egypt; 9 Yousef Abdullatif Jameel Chair of Prophetic Medical Applications (YAJCPMA), Faculty of Medicine, King Abdulaziz University, Jeddah 21589, Saudi Arabia; 10 Department of Human Anatomy and Embryology, Faculty of Medicine, Assiut University, Assiut 71515, Egypt; am.mahmoud@qu.edu.sa; 11 Department of Anatomy and Histology, College of Medicine, Qassim University, Buraydah 52571, Saudi Arabia

**Keywords:** immunity, chronic stress, antioxidant, pumpkin, glucocorticoid receptor, adrenergic receptor, TNF-α, IL-6

## Abstract

**Objectives**: Recently, increased attention has been given to pumpkin due to its proved nutritional components, which include antioxidant, antifatigue, and anti-inflammatory effects. The aim of the present work was to assess the impact of *Cucurbita pepo* L. (*CP*) on chronic unpredictable mild stress (CUMS)-induced changes in lymphoid organs through evaluating its effect on the histological structure of spleen, thymus gland, and lymph nodes compared to the antidepressant fluoxetine (FLU). **Materials and Methods**: Fifty male albino rats equally distributed into five groups that included control, control + *CP*, CUMS-exposed, FLU-treated, and *CP*-treated groups were used in this study. Rats were exposed to CUMS for 4 weeks, and treatment (either with FLU or *CP*) was started after 14 days of exposure. Behavior of the rats, serum corticosterone, oxidants/antioxidants profile, proinflammatory cytokines, and gene expression of glucocorticoid receptor (GR) and β-adrenergic receptor (β2-AR) were assessed after 28 days. Spleen, thymus gland, and lymph nodes were histopathologically assessed. **Results**: *CP* administration significantly reduced the CUMS-induced behavioural changes evident by the significant reduction in immobility time (*p* = 0.02) and corticosterone level (*p* < 0.001). Biochemically, *CP* reduced TNF-α and IL-6 (*p* < 0.001) and markedly alleviated the changes in oxidants/antioxidants in the serum and lymphoid organs compared to fluoxetine. *CP* significantly (*p* < 0.001) reduced CUMS-induced changes in GR and (β2-AR). Histopathologically, *CP* alleviated changes observed in the spleen, lymph nodes, and thymus gland. It significantly reduced the number of CD4, CD8, CD68, CD20, and caspase-3 immunopositive cells in the studied organs. **Conclusions**: This study proved the potential efficacy of *CP* in alleviating depression-associated immunodysregulation either alone or in combination with antidepressant therapy.

## 1. Introduction

The hypothalamic–pituitary–adrenal (HPA) axis plays a central role in the physiological stress response, including mood and immune regulation. HPA axis hyperactivity could lead to immune activation [1].

Immune dysfunction is considered a characteristic feature of multiple psychopathological conditions in humans, including depression, anxiety disorders, and chronic fatigue syndrome (CFS), which can be induced by chronic stress and endotoxin challenge [2,3]. It was reported that among the changes induced by exposure to chronic stress, immunoinflammation subsequently leads to psychiatric problems [4].

The immune system could be influenced by stress through stress hormones, such as catecholamines and glucocorticoids [5]. It was documented that stress changed the immune system via the glucocorticoid receptor (GR), which is expressed in immune cells, including T cells, B cells, monocytes, neutrophils, and macrophages [6,7]. Experiments on mice suggest that the increase of corticosteroids in response to stress suppresses the function of the different types of immune cells in the blood [8].

Few in vivo studies reported the effect of stress on lymphoid organs and showed alteration in histological architecture beside reduction in weight and cellularity. The thymus was the most sensitive organ among all the lymphoid organs that showed significant alterations after exposure to stress [9]. Spleen, the largest peripheral immune organ, showed chronic stress-induced alterations mediated by sympathetic and glucocorticoid actions [6,10], as well as cellular dysfunctions mediated through oxidative damage [11]. It was reported that the mRNA level of GR and β-adrenergic receptor (β2-AR) in immune cell subsets in the spleen was increased after 21 days of repetitive restraint stress (RRS) [10].

Fluoxetine (FLU), a classical antidepressant available in the market, was reported to induce neuroendocrine disruption [12]. Therefore, safer antidepressants with no or few side effects on the neuroendocrine system are needed.

Among the antidepressant foods that were studied by LaChance and Ramsey, pumpkin seeds proved to have an antidepressant food score of 47% [13]. Recently, seed extract of *Cucurbita pepo* L. (*CP*) was proved to be a possible “natural psychotherapeutic agent”, as it has a potent antioxidant and antidepressant effect [14]. In addition, the immunomodulatory effect of *CP* was also reported [15]. This study investigated the comparative immune effects of *CP* with fluoxetine in a multifaceted manner for the first time in a chronic stress model. It was conducted to assess the immunomodulatory effect of *CP* on immune cells residing in the lymphoid organs, spleen, and thymus of rats with chronic stress-induced depressive-like behavior.

## 2. Results

### 2.1. Behavioral Assessment

**Figure 1:**
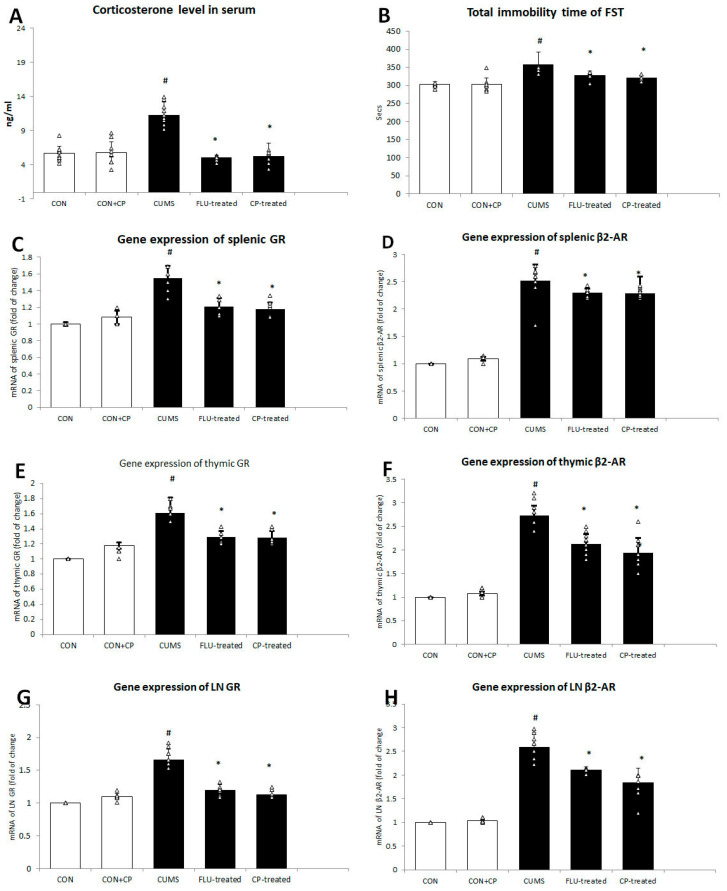
The effect of CUMS on corticosterone level in the serum (**A**) and total immobility time (**B**) of forced swimming test, gene expression of GR, and β2-AR in spleen (**C**,**D**) and thymus (**E**,**F**) and lymph node (**G**,**H**) assessed using quantitative real-time PCR (qRT-PCR). Data are presented in the form of mean ± standard deviation (SD). The F-test (one-way analysis of variance) was used to compare the studied groups followed by the post-hoc Bonferroni test. Significance was considered when *p* < 0.05. # significance versus control group. * significance versus CUMS group.

A significantly prolonged immobility time of FST (*p* ˂ 0.001) was observed in the CUMS group compared to the CON group, while it was significantly shorter in both Fluoxetine (FLU)-treated (*p* = 0.01) and *Cucurbita pepo* L. (*CP*)-treated (*p* = 0.001) groups compared to that of the CUMS group. No significant difference (*p* = 0.75) in the immobility time was noticed between the two groups (Figure 1).

### 2.2. Biochemical Assessment

#### 2.2.1. Serum Corticosterone Level

The level of corticosterone in the serum was significantly increased (*p* < 0.001) in the CUMS group compared to the CON group, while it was significantly reduced in FLU- (*p* = 0.01) and *CP*-treated (*p* = 0.001) groups when compared to CUMS group. No significant difference was notable in corticosterone level between FLU- and CP-treated groups (Figure 1).

#### 2.2.2. Gene Expression of GR and β2-AR in the Lymphoid Organs

Using qRT-PCR, gene expression of GR in splenic, thymic, and lymph node was found to be significantly increased (*p* < 0.001) in the CUMS group compared to the CON group, whereas it showed a significant decrease (*p* < 0.001) in FLU- (*p* < 0.001) and CP-treated (*p* < 0.001) groups when compared to the CUMS group (Figure 1).

Similar changes were observed in gene expression of β2-AR in spleen, thymus, and lymph node. It was significantly increased (*p* < 0.001) in the CUMS group compared to the CON group, whereas it showed a significant reduction in FLU- (*p* = 0.02, *p* < 0.001, *p* < 0.001) and CP-treated groups (*p* = 0.01, *p* < 0.001, *p* < 0.001), respectively, compared to the CUMS group (Figure 1).

#### 2.2.3. Inflammatory Cytokines in Serum and the Lymphoid Organs

**Figure 2:**
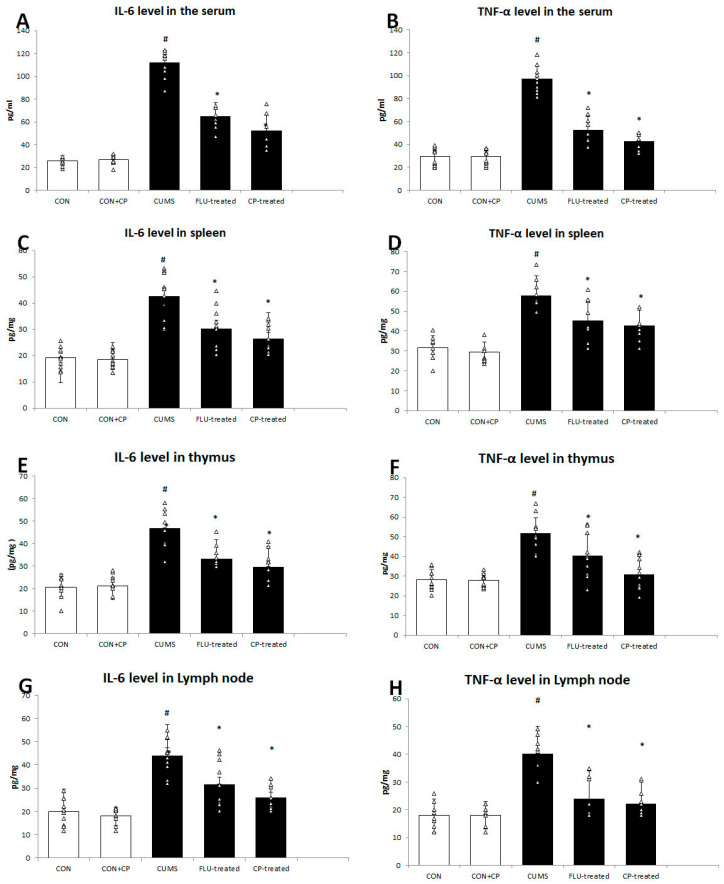
The effect of CUMS on the level of IL6 and TNF-α in the serum (**A**,**B**), spleen (**C**,**D**), thymus (**E**,**F**), and cervical lymph node (**G**,**H**). Data are presented in the form of mean ± standard deviation (SD). The F-test (one-way analysis of variance) was used to compare the studied groups followed by the post-hoc Bonferroni test. Significance was considered when *p* < 0.05. # significance versus control group. * significance versus CUMS group.

Serum levels of TNF-α, IL-6 were significantly increased (*p* < 0.001) in the CUMS group compared to the CON group, while they were significantly reduced (*p* < 0.001) in the FLU- and CP-treated groups compared to CUMS group with no significant differences (*p* = 0.15, *p* = 0.09) between both treated groups (Figure 2).

Splenic levels of TNF-α, IL-6 were also found to be significantly elevated (*p* < 0.001) in the CUMS group compared to the CON group, while they showed a significant reduction in the FLU- (*p* = 0.01, *p* = 0.004) and CP-treated (*p* = 0.001, *p* < 0.001) groups compared to the CUMS group (Figure 2).

In the same pattern, thymic and lymph node levels of TNF-α, IL-6 were significantly high (*p* < 0.001) in the CUMS group compared to the CON group, whereas they were significantly decreased in the thymus (*p* = 0.04, *p* = 0.001) and lymph node (*p* = 0.002, *p* = 0.001) of the FLU-treated group, as well as the thymus and lymph node (*p* < 0.001) of CP-treated groups, respectively (Figure 2).

#### 2.2.4. Assessment of the Levels of Oxidant/Antioxidants in the Serum and Studied Organs

A significant increase (*p* < 0.001) in the serum level of MDA was observed in the CUMS group compared to the CON group, while it was significantly reduced (*p* = 0.001) in FLU-treated and CP-treated groups compared to the CUMS group. No significant difference was notable in the serum MDA level in the CP-treated group compared to the FLU-treated group. Regarding MDA levels in spleen, thymus, and lymph node, it was significantly high (*p* < 0.001) in the CUMS group compared to the CON group, while it showed a significant reduction in FLU-treated and CP-treated groups compared to CUMS. CP significantly reduced MDA in the spleen, thymus, and lymph node compared to the FLU-treated group (Table 1 and Table 2).

A significant increase in level of MDA in the serum, spleen, thymus, and lymph node was observed in the CUMS group compared to the CON group, while it was significantly reduced in FLU-treated and CP-treated groups compared to the CUMS group. No significant difference was notable in the MDA level in the CP-treated group compared to the FLU- treated group (Table 1 and Table 2).

A notable reduction in SOD levels was recorded in the serum, spleen, thymus, and lymph node of the CUMS group compared to the CON group. Both FLU-treated and CP-treated groups showed a significant increase in SOD levels in serum, spleen, thymus, and lymph node compared to the CUMS group (Table 1 and Table 2).

Regarding the levels of CAT and GPX, it was found that CUMS induced a significant reduction in the serum, spleen, thymus, and lymph node compared to the CON group. FLU-treated and CP-treated groups showed a significant increase in CAT and GPX levels in serum, spleen, thymus, and lymph node compared to the CUMS group (Table 1 and Table 2).

### 2.3. Assessment of Splenic Parameters of the Studied Groups

**Figure 3:**
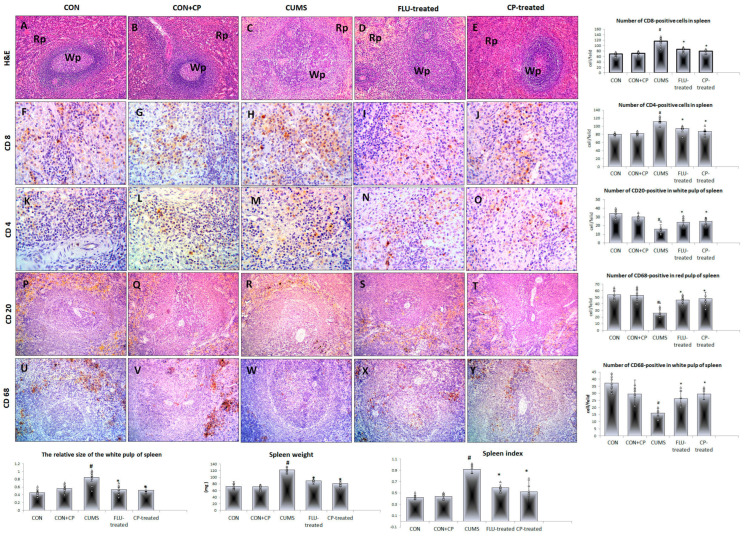
Photomicrographs of sections in the spleen of the studied groups. H&E-stained sections (**A**–**E**) show normal splenic architecture consists of white pulp (Wp) and red pulp (Rp) in both CON and CON + CP rats. White pulp is enlarged in CUMS group but appear nearly normal in FLU and CP-treated groups. Anti-CD8 (**F**–**J**), CD4 (**K**–**O**), CD-20 (**P**–**T**), and CD-68 (**U**–**Y**) immunostained sections of spleen are presented in the studied groups that included; CON & CON + CP, CUMS, FLU-treated, and CP-treated groups. (Magnification (**A**–**E**) and (**P**–**Y**) ×200, (**F**–**O**) ×400). Histograms show spleen weight, index, and the number of immunostained cells. Data are presented in the form of mean ± standard deviation (SD). The F-test (one-way analysis of variance) was used to compare the studied groups followed by the post-hoc Bonferroni test. Significance was considered when *p* < 0.05. # significance versus control group. * significance versus CUMS group.

A significant increase (*p* ˂ 0.001) in spleen weight and index was observed in CUMS compared to the CON group, while they were significantly reduced (*p* ˂ 0.001) in both FLU-treated and CP-treated groups compared to CUMS (Figure 3).

The spleen of rats of both CON and CON + CP groups showed normal splenic architecture and parenchyma that included the white and red pulp. The spleen of the CUMS group showed a significant increase (*p* = 0.003) in the relative size of the white pulp compared to the CON group, while it showed a significant reduction in FLU-treated (*p* = 0.04) and CP-treated (*p* = 0.03) groups compared to the CUMS group with no significant difference (*p* = 0.79) between the two treated groups (Figure 3).

Regarding the cellular subsets of the spleen, numerous CD8- and anti-CD4 positive cells were observed in the white pulp of the CON and CON + CP groups. A significant increase (*p* ˂ 0.001) in these cells was observed in the white pulp of spleen of CUMS compared to the CON group, while it was significantly reduced (*p* = 0.001) in FLU-treated and CP-treated groups compared to CUMS. Non-significant difference (*p* = 0.61, *p* = 0.91) was noted in their number when CP-treated groups were compared to the FLU-treated one, respectively (Figure 3).

Numerous CD20-positive cells and CD68-positive cells were observed in the white pulp and red pulp of the spleen, respectively, of both CON and CON + CP groups. The number of these cells showed a significant decrease in these areas (*p* ˂ 0.001) in the CUMS group when compared to the CON group, while it was significantly increased in FLU-treated (*p* = 0.001, *p* ˂ 0.001) and CP-treated (*p* ˂ 0.001) groups (Figure 3).

### 2.4. Assessment of Thymic Parameters of the Studied Groups

A significant decrease (*p* ˂ 0.001) in the thymus weight and index was observed in the CUMS group compared to the CON group, while they were significantly increased in FLU-treated (*p* = 0.04, *p* = 0.002) and CP-treated (*p* = 0.01, *p* ˂ 0.001) groups, respectively, when compared to the CUMS group.

**Figure 4:**
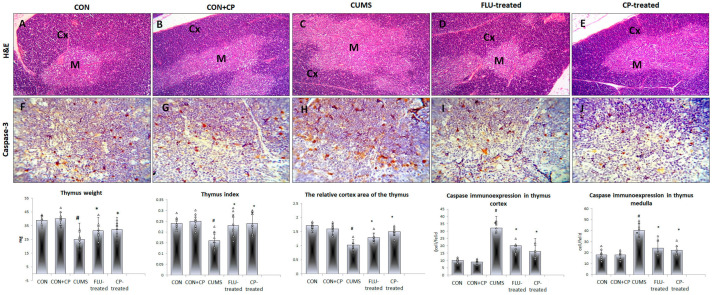
Photomicrographs of sections in the thymus of the studied groups. H&E-stained sections (**A**–**E**) show normal thymic lobules with outer dark cortex (Cx) and an inner light medulla (M) in CON and CON + CP rats. Widened medullary and retracted cortical tissue is observed in CUMS group. On the other hand, the normal architecture is preserved in FLU- and CP-treated groups. Anti-caspase 3 immunostained sections of the thymus (**F**–**J**) are presented in the studied groups that included CON & CON + CP, CUMS, FLU-treated, and CP-treated groups. (Magnification of (**A**–**E**) ×200, (**F**–**J**) ×400). Histograms show thymus weight, index, and the number of immunostained cells. Data are presented in the form of mean ± standard deviation (SD). The F-test (one-way analysis of variance) was used to compare the studied groups followed by the post-hoc Bonferroni test. Significance was considered when *p* < 0.05. # significance versus control group. * significance versus CUMS group.

Routinely stained sections of the thymus showed intact thymic lobules with outer dark cortex and inner light medulla in CON and CON-CP groups, while retracted cortical thymic tissue was observed in the CUMS group. Quantitative assessment revealed a notable decrease (*p* ˂ 0.001) in the relative cortex area of thymus in CUMS group compared to the CON group, while it was significantly increased (*p* ˂ 0.001) in both FLU-treated and CP-treated groups compared to CUMS. A significant increase (*p* = 0.01) in this parameter was notable in the CP-treated group compared to the FLU-treated group (Figure 4).

Assessment of apoptosis in the thymus of the studied groups using immunohistochemical technique revealed a significant increase in Caspase3-positive cells in the cortex and medulla of the CUMS group compared to the CON group, whereas their number significantly decreased (*p* ˂ 0.001) in FLU- and CP- treated groups compared to the CUMS group (Figure 4).

### 2.5. Assessment of Cervical Lymph Node Parameters of the Studied Groups

A significant decrease (*p* ˂ 0.001) in the weight of the cervical lymph node was observed in the CUMS group compared to the CON group, whereas a significant increase was noted in FLU-treated (*p* = 0.01) and CP-treated (*p* ˂ 0.001) groups when compared to CUMS.

**Figure 5:**
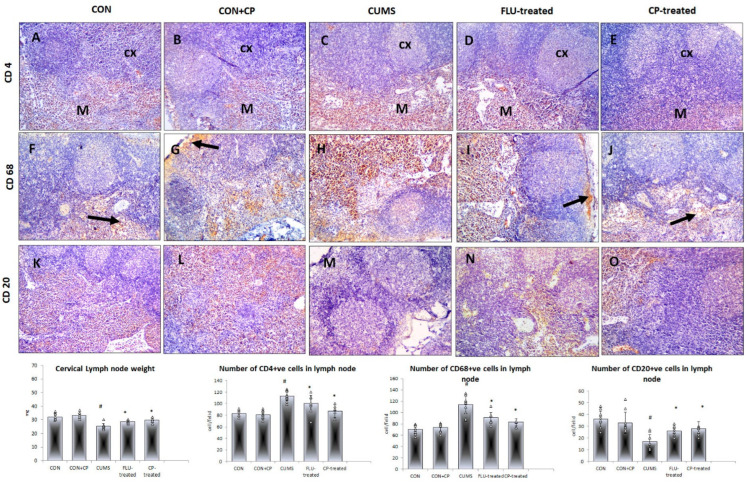
Photomicrographs of sections in the lymph node of the studied groups. Anti-CD4 (**A**–**E**) (arrow indicates the positive cells), CD-68 (**F**–**J**), and CD-20 (**K**–**O**) immunostained sections of lymph nodes are presented in the studied groups that included CON & CON + CP, CUMS, FLU-treated, and CP-treated groups. (Magnification ×200). Histograms show lymph nodes weight and the number of immunostained cells. Data are presented in the form of mean ± standard deviation (SD). The F-test (one-way analysis of variance) was used to compare the studied groups, followed by the post-hoc Bonferroni test. Significance was considered when *p* < 0.05. # significance versus control group. * significance versus CUMS group.

Few CD4- and CD68-positive cells were observed mostly in the cortex and medulla of the lymph node in both CON and CON + CP groups. The number of these cells was notably increased (*p* ˂ 0.001) in the cortex and medulla of CUMS group compared to CON group, whereas it was significantly reduced in FLU- (*p* = 0.04, *p* ˂ 0.001) and CP-treated (*p* ˂ 0.001) groups compared to the CUMS group, respectively (Figure 5).

On the other hand, lymph node showed many CD20-positive cells in the medulla of both CON and CON + CP groups with their number significantly (*p* ˂ 0.001) decreased in the CUMS group compared to the CON group, whereas it was significantly increased in both FLU- (*p* = 0.03) and CP-treated (*p* = 0.01) groups compared to the CUMS group (Figure 5).

## 3. Discussion

It was reported that stress either decreases or increases the immune function, depending on its duration, severity, and the associated psychological stress in both humans and animals [16]. Acute stress was proved to enhance the immune system, while chronic stress has a suppressive effect [17]. Pumpkin has obtained notable attention due to the health benefits attributed to its bioactive components. It was reported to decrease the depressive-like symptoms in rats, as was induced by imipramine [18]. Hence, in this work, the ameliorative effect of pumpkin was investigated on the dysregulated immunity in the peripheral tissues induced by exposure to chronic stress.

In this study, the depressive-like status of rats exposed to CUMS was documented by the significant increase of the immobility time of the FST and the significant increase in corticosteroid levels. Previously, it was reported that activation of the HPA is considered a physiological response of vertebrates associated with a significant increase in the glucocorticoid levels to cope with chronic stress [19]. The immunoregulation of corticosteroids is facilitated by specific binding of glucocorticoid to their receptors that are expressed in all leukocytes [20]. Increased level of glucocorticoids is considered a characteristic mediator during chronic stress inducing a negative immune response [21].

In the present work, it was observed that the serum and tissue levels of MDA, an indicator of lipid peroxidation, were elevated, while levels of SOD, GPX, and CAT were reduced in the CUMS-exposed rats. These findings were in agreement with those of Gavrilović and his colleagues who studied the changes in splenic tissue after different models of stress [22]. Increased MDA levels were attributed to changes in the lipid matrix and cell membranes that are indirectly induced by increased reactive oxygen species (ROS) [23].

In the current study, exposure to CUMS significantly increased the spleen weight and index and expanded the relative white pulp size. This is in agreement with some previous studies conducted on chronic stress [24,25,26]. One of these studies proved a significant role of oxidative stress in chronic stress-associated splenomegaly [27]. Exposure to chronic stress increased the size of red and white pulp of the spleen and subsequently disturbed the normal structure of the germinal center and resulted in splenomegaly [10,28]. On contrast with this finding, few studies have reported a decrease in the weight of spleen following exposure to some types of stress [29].

Chronic stress, in this study, resulted in a change in the splenic cellularity where the total CD4-, CD8-positive T cells were significantly increased, while the splenic CD68-positive macrophages and CD20-positive pro B cells are significantly reduced. In agreement with this, Gurfein et al. reported that exposure of mice to chronic mild stress changed the ratio between B and T lymphocytes in the spleen as a result of reduction in the number of B cells and increase in the number of T cells [30]. More recent, splenic T lymphocyte populations were reported also to be increased upon exposure of a mouse model of CUMS coupled with lipopolysaccharide (LPS) [26] and repetitive restrain stress (RRS) [10]. Li et al. added that during exposure to RRS immune cells, including B lymphocytes, macrophages were reduced in the spleen and were redistributed to the peripheral blood [10]. The ratio of T helper cells to cytotoxic T cells is known as the CD4+/CD8+ ratio, which has been used as a clinical index to evaluate patient’s immunity. A reduction in this ratio indicates “immunosenescence” and an inability to resist infection [31,32,33].

Most immune cells such as T cells, B cells, and macrophages were described to express GR, β-AR [7]. It was also reported that stress changed the immune system via GR [6]. In this study, exposure to CUMS induced a significant expression of GR, β-AR in the lymphoid organs. In concordance with these findings, Li et al. found that the expression of both β-AR and GR of immune cell subsets in the spleen was up-regulated after exposure to RRS, and this might be behind the recoded changes in these immune cells [10].

In this study, CUMS exposure resulted in reduced thymus weight and index, retracted cortical tissue, and reduced weight of lymph node. These findings were in line with those previously reported in mice exposed to CUMS coupled with LPS [26]. Reduced weight of thymic gland could be attributed to increased apoptosis confirmed immunohistochemical using caspase-3. Apoptosis of thymocytes might be attributed to increased corticosterone in the serum after CUMS exposure. It was frequently reported that high levels of corticosterone cause apoptosis of immune cells [9,34].

A significant crosstalk exists between stress and ferroptosis, a newly identified form of regulated cell death that is driven by the accumulation of lipid peroxides and is characterized by the loss of membrane integrity [35,36]. Ferroptosis can be induced by stressors through multiple mechanisms and can exacerbate cellular damage and death, contributing to the progression of stressful disorders [36]. Therefore, ferroptosis might be involved in chronic stress and the associated changes observed in the lymphoid organs.

Among the important findings noted in this study is the disturbed cytokine levels (TNF-α and IL-6) either in the serum or the studied lymphoid organs following exposure to CUMS. The pro-inflammatory cytokines IL-6 and TNF-α were increased in depressed patients and were found to disturb the immune cells and “the neuroendocrine path”, which subsequently activate the HPA axis [37]. Disturbed cytokine levels might be behind the disrupted immune cell responses and increased apoptosis of T cell and dendritic cell reported after chronic stress conditions in some previous studies [16,38].

Administration of pumpkin extract, in this study, was associated with amelioration of the depressive behavior as evidenced by decreased immobility time of the FST, reduced corticosterone, and inflammatory cytokines TNF-α and IL-6 in the serum. A reduction of the level of inflammatory cytokines in the depressed rats was previously confirmed after administration of Sweetme Sweet Pumpkin (SSP) for 28 days [39]. They added that oleic and palmitic acids, as well as estradiol, the active compounds present in pumpkin, may be responsible for its anti-inflammatory activity.

Interestingly, both FLU- and CP-treated groups showed a significant increase in SOD, GPX, and CAT levels in the serum, as well as in the spleen, thymus, and lymph node in a comparable pattern. Some researchers have confirmed that polysaccharides present in pumpkin inhibit LDL oxidation [40]. It has been reported that enzymatic and non-enzymatic antioxidants form a complex defense network that maintains redox balance in the bloodstream, which is crucial in preventing oxidative stress-related diseases and preserving cellular health. This antioxidant system is particularly relevant in the context of ferroptosis [41].

Because of the biologically active components such as polysaccharides, proteins, peptides, sterols, fixed oils, and para-aminobenzoic acid, pumpkin is considered a medicinal plant that is used as “functional food” promoting health status when safely consumed [42]. In this study, the CP-treated group showed a significant reduction in both CD4 and CD8 numbers, while the B cells and CD68 number were significantly increased compared to the CUMS group. In line with this, pumpkin seed oil extracts were proved to have immunomodulatory effects [15]. Huang et al. reported that the immunomodulatory effect induced by pumpkin on macrophages was due to its neutral polysaccharide [43]. In patients with depression, the level of serotonin and norepinephrine, the hormones responsible to cure depression, was increased in the brain when treated with pumpkin and β carotene [39].

Our finding about the antiapoptotic effect of pumpkin extract was supported by a previous study, which showed that the oral administration of pumpkin seed oil (PSO) alleviated alternations induced by methotrexate through the potent antioxidant, anti-inflammatory, and anti-apoptotic activity of pumpkin constituents such as linoleic acid and Omega-3 [44].

## 4. Materials and Methods

The primary assessed outcome, in this study, was to evaluate the immunomodulatory effects of *Cucurbita pepo* L. on immune cells residing in the lymphoid organs, spleen, and thymus, while the secondary assessed outcome was to assess the anti-inflammatory and antioxidant effects of *Cucurbita pepo* L. and explore the mechanism behind its effects.

### 4.1. Preparation of Cucurbita pepo Extract

Fresh pumpkin (*Cucurbita pepo* L.) CP was purchased from the local market at Jeddah, Saudi Arabia and was identified by a specialist at the Botany department, Faculty of Science, King Abdulaziz University (KAU). Preparation of pumpkin extract was explained in a previous work [45].

As was previously described, the ethanolic extract of CP was prepared from its dried powder (50 g) [46]. The yield of extraction was 41%. After being dissolved in distilled water, CP extract was stored in a suitable container until use. CP extract dissolved in distilled water at a dose of 100 mg/kg once daily by gavage for 2 weeks. This dose was proved to induce antifatigue, ref. [47] antioxidant, and antidepressant effects [48].

Fluoxetine (FLU) (Dar Al Dawah Pharmaceuticals Co., Ltd., Amman, Jordan), an antidepressant that is used in this study for pharmacological validation, was dissolved in 0.03% carboxymethyl cellulose (CMC-Na) at a dose of 20 mg/kg by gastric gavage, as was previously reported [49].

### 4.2. Study Protocol

All the animal experiments were conducted according to the institutional guidelines for animal experiments, and the study protocol by the biomedical research ethics committee at the Faculty of Medicine, King Abdulaziz University (KAU), Jeddah, KSA (reference number 45-20) on 8 January 2020.

CUMS procedure used in this study included exposure of animals to different types of stressors at different times during the day for 4 weeks in order to prevent habituation to stress as was previously described [50]. Stressors included social stress by placing mice in soiled cages of other mice, inversing the light/dark cycle, placing mice in cages with wet sawdust, tilting cages to 30_ and restraining the mice, and water stress by placing mice in an empty cage with 1 cm of water at the bottom.

Fifty adult male albino rats with average weight (150–200 g) and average age (2–3 months) were used in this study. They were purchased from KFMRC and left for 7 days to acclimatize to the laboratory conditions before starting the experiment. The rats were randomly distributed into five groups. One control group (CON) was left unexposed to CUMS and another control group (CON + CP) was left without exposure to stress and received CP for 4 weeks. The other three groups were exposed, respectively, to CUMS and 0.03% CMC-Na (CUMS group), CUMS and FLU (FLU-treated group), and CUMS and CP (CP-treated group). All treatments started after 2 weeks of exposure to CUMS and continued for 2 weeks.

### 4.3. CUMS-Induced Behavioral Changes

Using the forced swim test (FST), the depressive-like behavior of the rats was assessed after exposure to CUMS, as described in previous studies [51]. The total immobility time was recorded and expressed in seconds.

### 4.4. CUMS-Induced Biochemical Changes

In the morning after 4 weeks of CUMS exposure, the mice received 4% isoflurane and blood samples were drawn from the retroorbital venous plexus and placed in EDTA-coated tubes. Blood samples were centrifuged for 10 minutes, and the serum was kept at 80 °C.

Serum corticosterone (ALPCO Diagnostics, Orangeburg, NY, USA), tumor necrosis factor-α (TNF-α), and interleukin (IL-6) (quantakin R & D system, USA Kit, R & D system, Minneapolis, MN, USA) levels were assessed at the end of the 4 weeks using enzyme-linked immunosorbent assay (ELISA) kits.

Malondialdehyde (MDA), Superoxide dismutase (SOD), catalase (CAT) (Biodiagnostic, Egypt), and Glutathione peroxidase (GPX) (Randox Labs, Crumlin, UK) levels were assessed in the serum and lymphoid organs using assay kits based on the method previously described [52,53].

Quantitative Realtime polymerase chain reaction (qRT-PCR) was used for assessment of splenic, thymic, and lymph node gene expression of GR and β2-AR. After deparaffinization of formalin-fixed paraffin-embedded (FFPE) sections of spleen, thymus, and cervical lymph node with 1 mL of xylene, the RNA was extracted from 100 mg of these sections using Trizol according to the supplier instruction (Invitrogen Life Technologies, Carlsbad, CA, USA).

Genomic DNA contamination was removed from RNA samples by ribonuclease inhibitor and NanoDrop 2000 Spectrophotometer (Thermo Scientific, Waltham, MA, USA) was used to ensure purity of extracted RNA. Complementary DNA (cDNA) was synthesized as described by Bunker et al. [54] using the Revert Aid First Strand cDNA Synthesis Kit. The cDNAs obtained were amplified by using PCR Master Mix (Bioneer, Daejeon, Republic of Korea) with primers designed by Metabion International (Semmelweisser, Germany) as follows: GR (TCTCAGGCAGATTCCAAGCA-TGGACAGTGAAACGGCTTTG)

β-adrenergic receptor (β2-AR) (CACAGCCATTGCCAAGTTC- CGGGCCTTATTCTTGGTCAGC) and β-actin (forward 5′-TCTGGCACCACA CCTTCTA-3; reverse 5′-AGGCATACAGGGACAGCAC-3). The assay was done as defined [54].

### 4.5. Histopathological Assessment

Rats of all groups were sacrificed, after 4 weeks, by cervical decapitation. Incision of chest and abdomens were done. Spleen, thymus gland, and cervical lymph nodes were rapidly dissected out and fixed in 10% neutral buffered formalin overnight, then processed to obtain paraffin blocks. Serial paraffin sections were cut into 3–4 μm thickness and stained with hematoxylin and eosin (H & E) for the histopathological and immunohistochemical assessment [55].

### 4.6. Immunohistochemical Studies

The paraffin sections were deparaffinized, rehydrated, and boiled in 0.01 M of sodium citrate buffer (pH: 6) in a microwave for 20 min for antigen retrieval. To block endogenous peroxidase activity, 3% H_2_O_2_ in methanol was used for 5 min at room temperature, followed by washing twice in phosphate-buffered saline (PBS).

The primary mouse anti-CD68 monoclonal antibody (Dako A/S DK-2600, Glostrup, Denmark, dilution 1/100), anti-CD3 and anti-CD8 (Novocastra Laboratories Ltd., Newcastle, UK), and anti CD20 (BioCare Medical, Pachieco, CA, USA, at a dilution 1/100) were put on the sections overnight at room temperature, then exposed to biotinylated goat anti-rabbit IgG and streptavidin peroxidase complex (1:200 dilution; Vector Laboratories, Newark, CA, USA) at room temperature. After washing, the slides were incubated with avidin–biotin–peroxidase complexes (Dako-USA, Carpinteria, CA, USA) for 10 min, covered by DAB and incubated for 10 min, then counterstained with hematoxylin.

Negative control sections were obtained by omitting the primary antibody during staining of some slides. The nuclei were counterstained with hematoxylin. Brown cytoplasmic staining was considered positive reaction. The stained slides were blindly examined by a histopathologist.

Photographing of stained slides was done by using “Olympus Microscope BX-51 provided with a digital camera”. Pro Plus image analysis software version 6.0 was used for assessment of number of CD68-positive groups.

### 4.7. Statistical Analysis

The raw data of this study were analyzed using Statistical Package of Social Science Program (SPSS 2016, SPSS Inc., Chicago, IL, USA). The results were presented as mean ± standard deviation. The F-test (one-way analysis of variance), followed by the post-hoc Bonferroni test was used to compare each two groups and avoid repeated comparisons. Significance was considered at *p* < 0.05. The sample size was determined using power analysis. The effect size was 20%, the confidence level (alpha) was 0.05, and the sample power was 80% (https://clincalc.com/stats/samplesize.aspx, accessed on 1 June 2023). The experimental unit of the study was the rat. No inclusion or exclusion criteria were adopted regarding the animals. No experimental units were excluded during the analysis.

## 5. Conclusions

The behavioural, biochemical, and histopathological results of this study coincided together to prove that pumpkin has anti-inflammatory, antioxidant, antiapoptotic, and immunomodulatory effects. Therefore, it is considered an excellent medicinal plant that possesses a considerable number of beneficial components. These findings suggest that pumpkin extract may have potential as an adjunctive agent for managing stress-induced immune dysregulation.

## Figures and Tables

**Table 1:** Effect of treatment on oxidants/antioxidants profile in the serum of the studied groups.

	CON (Mean ± SD)	CON + CP	CUMS	FLU-Treated	CP-Treated
Serum					
SOD (u/mL)	18.91 ± 2.92	19.27 ± 3.68 *p* = 0.344	9.9 ± 2.1 *p* < 0.001	14.74 ± 3.7 P1 = 0.03	15.67 ± 3.8 P1 = 0.004 P2 = 0.51
GPX (u/mL)	58.60 ± 7.76	60.67 ± 13.23 *p* = 0.56	37.5 ± 4.9 P1 < 0.001	50.39 ± 10.6 P1 = 0.03	51.92 ± 6.15 P1 = 0.01 P2 = 0.61
CAT (u/mL)	0.41 ± 0.09	0.44 ± 0.07 *p* = 0.31	0.24 ± 0.06 P1 < 0.001	0.36 ± 0.10 P1 = 0.01	0.39 ± 0.08 P1 = 0.002 P2 = 0.81
MDA (nmol/mL)	1.35 ± 0.14	1.37 ± 0.25 *p* = 0.15	2.23 ±0.71 P1 < 0.001	1.59 ± 0.37 P1 = 0.001	1.51 ±0.18 P1 = 0.001 P2 = 0.86

The results were presented as mean ± standard deviation (SD). The F-test (one-way analysis of variance), followed by the post-hoc Bonferroni test, was used to compare each two groups. Significance was considered at *p* < 0.05. *p* value of comparison of this group versus the control. P1 value of comparison of this versus CUMS group. P2 value of comparison of this versus FLU-treated group. CON = control. CUMS = chronic Unpredictable Mild Stress.

**Table 2:** Effect of treatment on oxidants/antioxidants profile in the lymphoid organs of the studied groups.

	CON (Mean ± SD)	CON + CP	CUMS	FLU-Treated	CP-Treated
Spleen					
SOD (u/mg protein)	4.45 ± 0.72	4.59 ± 1.10 *p* = 0.26	1.84 ± 0.61 *p* < 0.001	3.09 ± 0.82 P1 = 0.03	4.42 ± 1.03 P1 < 0.001 P2 = 0.02
GPX (nmol/mg protein)	58.93 ± 5.13	57.41 ± 7.29 *p* = 0.78	35.71 ± 12.51 *p* < 0.001	49.37 ± 9.26 P1 = 0.02	52.76 ± 9.21 P1 = 0.001 P2 = 0.23
CAT (u/mg protein)	123.17 ± 10.31	121.33 ± 12.81 *p* = 0.83	83.87 ± 13.23 *p* < 0.001	101.13 ± 13.73 P1 = 0.03	115.14 ± 10.66 P1 < 0.001 P2 = 0.14
MDA (u/mg protein)	11.46 ± 0.98	12.28 ± 1.43 *p* = 0.81	20.24 ± 3.18 *p* < 0.001	16.19 ± 1.4 P1 < 0.001	12.82 ± 3.08 P1 = 0.003 P2 = 0.02
Thymus					
SOD (u/mg protein)	4.61 ± 1.38	4.39 ± 1.41 *p* = 0.55	1.31 ± 0.57 *p* < 0.001	3.11 ± 1.01 P1 = 0.02	4.55 ± 1.39 P1 = 0.001 P2 = 0.10
GPX (nmol/mg protein)	63.32 ± 10.34	62.71 ± 9.50 *p* = 0.13	37.82 ± 11.88 *p* < 0.001	52.26 ± 7.57 P1 = 0.04	59.76 ± 12.31 P1 < 0.001 P2 = 0.43
CAT (u/mg protein)	117.78 ± 10.50	113.28 ± 12.01 *p* = 0.83	83.87 ± 15.09 *p* < 0.001	108.33 ± 13.26 P1 < 0.001	105.44 ± 8.02 P1 = 0.002 P2 = 0.91
MDA (u/mg protein)	13.69 ± 2.68	12.75 ± 2.32 *p* = 0.56	24.49 ± 4.12 *p* < 0.001	19.11 ± 2.48 P1 = 0.004	14.68 ± 3.53 P1 < 0.001 P2 = 0.03
Lymph node					
SOD (u/mg protein)	4.11 ± 0.68	4.49 ± 0.85 *p* = 0.65	1.90 ± 0.70 *p* < 0.001	3.22 ± 0.67 P1 = 0.004	3.98 ± 0.94 P1 < 0.001 P2 = 0.34
GPX (nmol/mg protein)	60.96 ± 7.87	56.52 ± 5.31 *p* = 0.32	34.21 ± 12.21 *p* < 0.001	50.16 ± 8.80 P1 = 0.002	51.35 ± 8.83 P1 = 0.001 P2 = 0.43
CAT (u/mg protein)	119.07 ± 8.30	117.23 ± 9.50 *p* = 0.62	83.37 ± 13.63 *p* < 0.001	98.02 ± 12.16 P1 = 0.04	113.4 ± 10.79 P1 < 0.001 P2 = 0.03
MDA (u/mg protein)	13.50 ± 2.35	12.01 ± 1.51 *p* = 0.91	22.99 ± 5.20 *p* < 0.001	17.89 ± 2.44 P1 = 0.001	12.76 ± 2.89 P1 < 0.001 P2 = 0.01

The results were presented as mean ± standard deviation (SD). The F-test (one-way analysis of variance), followed by the post-hoc Bonferroni test, was used to compare each two groups. Significance was considered at *p* < 0.05. *p* value of comparison of this group versus the control. P1 value of comparison of this versus CUMS group. P2 value of comparesion of this versus FLU-treated group. CON = control. CUMS = chronic Unpredictable Mild Stress.

## Data Availability

The original contributions presented in this study are included in the article. Further inquiries can be directed at the corresponding author.
